# Transcriptome Analysis of Neuroendocrine Regulation of Ovine Hypothalamus-Pituitary-Ovary Axis during Ovine Anestrus and the Breeding Season

**DOI:** 10.3390/genes12121861

**Published:** 2021-11-24

**Authors:** Yingjie Zhong, Ran Di, Yang Yang, Qiuyue Liu, Mingxing Chu

**Affiliations:** 1 Institute of Animal Sciences, Chinese Academy of Agricultural Sciences, Beijing 100193, China; zhongyingjie1996@163.com (Y.Z.); diran@caas.cn (R.D.); yy176362@163.com (Y.Y.); 2 Institute of Genetics and Developmental Biology, Innovation Academy for Seed Design, Chinese Academy of Sciences, Beijing 100101, China

**Keywords:** RNA-seq, sheep, seasonal estrus, hypothalamus, pituitary, ovary

## Abstract

Most sheep are seasonal estrus, and they breed in autumn when the days get shorter. Seasonal estrus is an important factor that affects the productivity and fertility of sheep. The key point to solve this problem is to explore the regulation mechanism of estrus in sheep. Therefore, in this study, transcriptomic sequencing technology was used to identify differentially expressed mRNAs in the hypothalamus, pituitary and ovary of Small Tail Han sheep (year-round estrus) and tan sheep (seasonal estrus) among luteal, proestrus and estrus stages. There were 256,923,304,156 mRNAs being identified in the hypothalamus, pituitary and ovary, respectively. Functional analysis showed that the photosensor, leucine and isoleucine biosynthesis pathways were enriched significantly. It is speculated that photoperiod may initiate estrus by stimulating the corresponding pathways in hypothalamus. *ODC1*, *PRLH*, *CRYBB2*, *SMAD5*, *OPN1SW*, *TPH1* are believed to be key genes involved in the estrogen process. In conclusion, this study expanded the database of indigenous sheep breeds, and also provided new candidate genes for future genetic and molecular studies on the seasonal estrus trait in sheep.

## 1. Introduction

The life activities of animals are greatly affected by seasonal changes. A common reproductive strategy among mammals living in a seasonally fluctuating environments is the restriction of breeding activity to an optimal time of year [1]. This helps to ensure that the offspring can be born in the best season for survival and growth [2]. Mammals usually rely on photoperiod to time their annual reproductive rhythm. Seasonal breeding animals are divided into short-day (SD) breeding animals and long-day (LD) breeding animals according to different responses of animal breeding activities to light cycles. Sheep, goats, deer, etc. are seasonal breeding animals and mate in autumn and winter when sunshine is gradually shortened, which is called “short-day animals”. Horses, hamsters, hedgehogs, weasels, etc. are estrous and mating in spring and summer when the sunshine is getting longer. They are called “long-day animals”. Those seasonal breeding animals can only estrus and ovulate during the estrous seasons. During the non-estrous season, the ovarian activities are at rest and, generally, the animal does not ovulate, which is called the anestrus period. During the estrous season, some animals have multiple estrous cycles. These animals called seasonal multiple estrus, such as horses, donkeys, sheep and goats. Some have only one estrous cycle during the estrus season, which is called seasonal single estrus. For example, dogs have two estrus seasons, namely spring and autumn, with only one estrous cycle per season [3]. Seasonal breeding is the main limiting factor to restrict the balanced supply throughout the year [4].

The reproductive system shows regular estrous cycles in mammals, and it is synchronized with external environmental factors such as seasons or photoperiods. Therefore, most species have evolved corresponding physiological regulation mechanisms in order to adapt to changes in the external environment [5]. Seasonal reproduction in mammals is regulated by the hypothalamus-pituitary-gonad axis (HPG) which is a process that needs to be coordinated by the nerve and endocrine systems. Hypothalamic neurons can synthesize and release decapeptide and gonadotropin releasing hormone (GnRH) into the hypothalamic portal blood system. GnRH stimulates the secretion of gonadotropins (luteinizing hormone (LH) and follicle stimulating hormone (FSH)) in the anterior pituitary to activate gonadal activity, but the release of GnRH is also regulated by gonadal hormone feedback [6]. The HPG axis of seasonal breeding animals is activated only during the breeding season [5]. Ernst and Berta Scharrer discovered neurosecretion in fish and insects as early as the 1920 years. A substance secreted by neurons that is carried to all parts of the body and produces effects, such as gonadotropin-releasing hormone (GnRH) controlling the release of luteinizing hormone (LH) [7]. In the 1940 years, Geoffrey Harris [8] of Cambridge University confirmed the existence of pituitary-portal circulation. He believes that substances produced by the hypothalamus stimulate the anterior pituitary to release various hormones to maintain the function of endocrine glands (thyroid gland, adrenal cortex, gonad). HPG axis plays a key role in controlling gonadal functions in mammals such as follicular development, ovulation, spermatogenesis, etc. [9]. In seasonal breeding sheep, the secretion of melatonin in the pineal gland is used to regulate the nerve LH pulse generator during the day, and the seasonal breeding state of ewes is determined by changing the frequency of GnRH pulse [10]. The positive feedback effect of estrogen leads to the surge of LH secretion in pituitary gland reaching the maximum before ovulation, which is related to the increase of GnRH secretion in sheep hypothalamus [11]. At the end of breeding, the peaks of GnRH and LH decreased to the lowest, making the sheep enter the period of rest. In anestrus season, hormone secretion is less, and GnRH and LH peaks also maintain at a low level. It has systematically been found that the transfer from SD to LD quickly prompts the arrest of cyclicity (approximately 30 days), whereas exposure of anestrous ewes (LD state) to SD elicits a return to cyclicity after a longer duration (often between 60 and 90 days) [10]. Studies listed above showed that the secretion of gonadotropin in sheep hypothalamus also has seasonal rhythm.

In this study, transcriptome sequencing technology and bioinformatics analysis were used to identify the differentially expressed mRNAs and their potential biological functions in sheep hypothalamus, pituitary gland and ovary at different stages (luteal stage, proestrus stage and estrus stage). Furthermore, the key genes involved in seasonality of sheep were identified to provide an important reference for the exploration of the estrus mechanism of sheep.

## 2. Materials and Methods

### 2.1. Experimental Animals, Tissue Collection and Hormone Determination

All experiments involving animals were authorized by the Science Research Department (in charge of animal welfare issue) of the Institute of Animal Sciences, Chinese Academy of Agricultural Sciences (IAS-CAAS; Beijing, China). They were authorized by the Animal Ethics Committee of the Institute of Animal Science, Chinese Academy of Agricultural Sciences (no. IAS2019-49).

**Figure 1:**
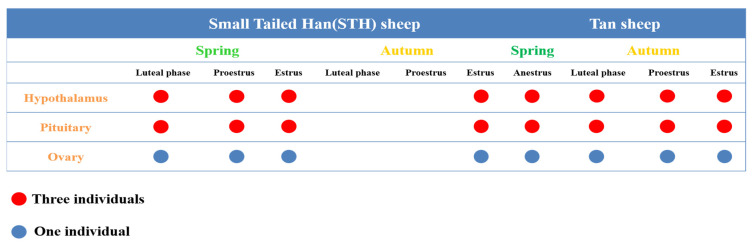
The number of hypothalamus, pituitary and ovary samples collected from 8 different reproductive stages. The stages including Tan ewes in spring at anestrous stages (TSA), Tan ewes in autumn at luteal phase (TAL), Tan ewes in autumn at proestrus stage (TAP), Tan ewes in autumn at estrus stage (TAE), STH ewes in spring at luteal phase (HSL), STH ewes in spring at proestrus (HSP), STH ewes in spring at estrus stage (HSE) and STH ewes in autumn at estrus stage (HAE).

All procedures involving animals are approved by the animal care and use committee of the institution in which the experiment is conducted. Tan sheep and Small Tail Han (STH) sheep were selected from the same breed protection farm in Ningxia Autonomous Region, China, and those raised under similar conditions with free access to water and food in natural lighting. In the four seasons of spring (March to May), summer (June to August), autumn (September to November) and winter (December to February of the following year), ewe of the 3-year-old, clinically normal and non-pregnant Tan sheep and STH sheep were tested daily for estrous activity. The test of estrous activity is to allow a ram wearing an isolate cloth with a strong sexual desire to show complete sexual behavior to the ewe, and the ewe is considered to be in estrous when they accept the crawling behavior of the ram. Ewe in estrus and ewe without estrus were selected according to the obvious signs of estrus induction. We recorded three consecutive estrous cycles per ewe, and plasma concentrations of progesterone and luteinizing hormone in ewe during the luteal and proestrus periods were measured to describe the specific stages of the estrous cycle. Finally, eight ewes representing eight different reproductive stages were randomly selected for ovarian collection (Figure 1). Meanwhile, we collected the hypothalamus and pituitary tissues of eight ewes at different reproductive stages, and collected three individual samples at each stage. All samples were immediately stored at −80 °C for total RNA extraction.

### 2.2. RNA Extraction, Library Preparation and RNA Sequencing

Total RNA was isolated from ovarian tissue with TRIzol reagent (Invitrogen Inc., Carlsbad, CA, USA) at different time periods according to the manufacturer’s directions. The mRNA libraries for different reproductive stages were generated using Illumina Truseq RNA Sample Preparation Kits. The required fragments were enriched by PCR amplification and purified using a Qiagen MiniElute PCR Purification Kit. The library products were sequenced with an Illumina HiSeq 2000 system and 100 bp paired-end reads were generated, which has been described in our previous work [12]. The library construction and sequencing of mRNAs was performed at Beijing Institute of Genomics, Chinese Academy of Sciences (Beijing, China). The sequence datasets have been deposited in the BioProject (Biological Project Library) database of Genome Sequence Archive [13] in National Genomics Data Center [14], Beijing Institute of Genomics (China National Center for Bioinformation), Chinese Academy of Sciences, with the accession number PRJCA000881.

### 2.3. Quality Control and Bioinformatics Analysis of Sequenced RNAs

For raw sequenced data (fastq format), quality control and sequence statistics were performed by FastQC version 0.10.1 (http://www.bioinformatics.babraham.ac.uk/projects/fastqc/ accessed on 30 June 2021). Clean data were obtained by removing reads containing adapter, reads on containing ploy-N and low-quality reads from raw data. Ovis aries reference genome and gene model annotation files were downloaded from genome website directly (Oar_v3.1). The spliced mapping process was performed by TopHat for each sample against to both genome and transcriptome references. Finally, SAMtools (version 0.1.18) and Linux Shell were used to extract mapped reads and other statistical information. Gene expression levels were calculated based on read counts which also included unambiguous mapped reads according to negative binomial distribution. RPKM (Reads perkilobase of exon model per million mapped reads) values represent expression level of both gene and transcript.

The differentially expressed mRNAs among different stages were obtained by the edgeR software package which used an empirical Bayesian method to test differential expression in deep sequencing datasets, and during this process the sequencing depth was normalized using edgeR software. A criterion of absolute log2 (fold change) > 1 and *p* < 0.01 was used to identify differentially expressed genes.

### 2.4. Gene Ontology and KEGG Pathway Analysis of Differentially Expressed Genes

The Gene Ontology (GO) and Kyoto Encyclopedia of Genes and Genomes (KEGG) enrichment analysis of DE mRNAs was performed using the online tool G: profiler [15] (releases/2020-10-20, https://biit.cs.ut.ee/gprofiler/gost accessed on 30 June 2021) to understand the biological function of DE mRNAs.

### 2.5. Integral Protein-Protein Networks Analysis

To clarify the potential relationships of differentially expressed (DE) mRNAs with different organizations at different periods, PPI network was constructed by the STRING [16] (Version 11.0b, releases/2020-10-17, https://string-db.org/ accessed on 30 June 2021) and visualized by the cytoscape (Version 3.4.0) [17]. In this network, nodes and edges represent biological data in a direct way, in which each node represents a biological molecule and the edges represent the interactions between nodes.

### 2.6. Statistical Analyses

Statistical analysis was conducted by SAS (v9.2). *p* ≤ 0.05 was consider as significant difference.

## 3. Results

### 3.1. Summary of RNA Sequencing Data

**Figure 2:**
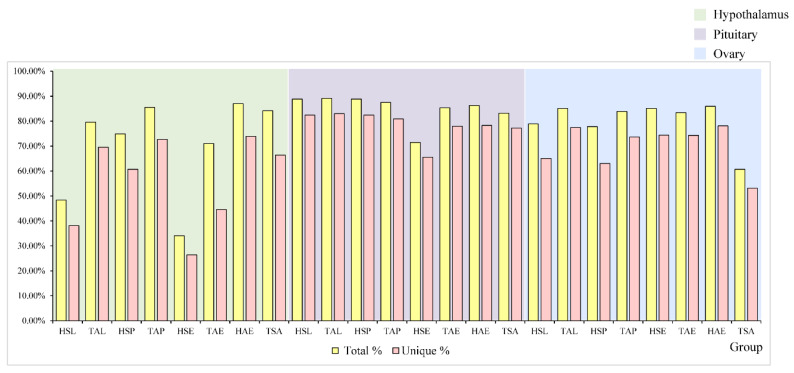
Total and unique mapped for each group in the hypothalamic, pituitary, and ovary.

To understand the expression pattern of mRNAs in anestrus and the three stages (luteal phase, proestrous and estrous) during the breeding season, 24 hypothalamus, pituitary and ovary samples from eight groups (TSA, TAL, TAP, TAE, HSL, HSP, HSE, HAE, *n* = 3) were sequenced separately. More than ten million raw reads were generated for each group respectively (Tables S1 and S2). The total mapped rate in individual sample was above 30% after data filtering. The unique mapped rate in individual sample was above 20% after data filtering (Figure 2).

### 3.2. Identification of DE mRNAs by RNA Sequencing

Sequencing analysis was carried out using mRNAs libraries derived from three tissues (hypothalamus, pituitary, ovary) of STH and Tan sheep at different stages (Luteal phase, Proestrus, Estrus). The expression profiles of anestrous Tan sheep were compared with those in the other three stages in both Tan and STH sheep breeds (Table 1 and Table 2). In hypothalamus, 21, 15 and 0 significantly DE mRNAs were identified between anestrus vs. luteal phase, anestrus vs. proestrus and anestrus vs. estrus, respectively. In pituitary, 51, 28 and 10 significantly DE mRNAs were identified between anestrus vs. luteal phase, anestrus vs. proestrus and anestrus vs. estrus, respectively. There were 315, 93 and 52 DE mRNAs identified between the anestrus vs. luteal phase, anestrus vs. proestrus and anestrus vs. estrus in ovary, respectively.

**Figure 3:**
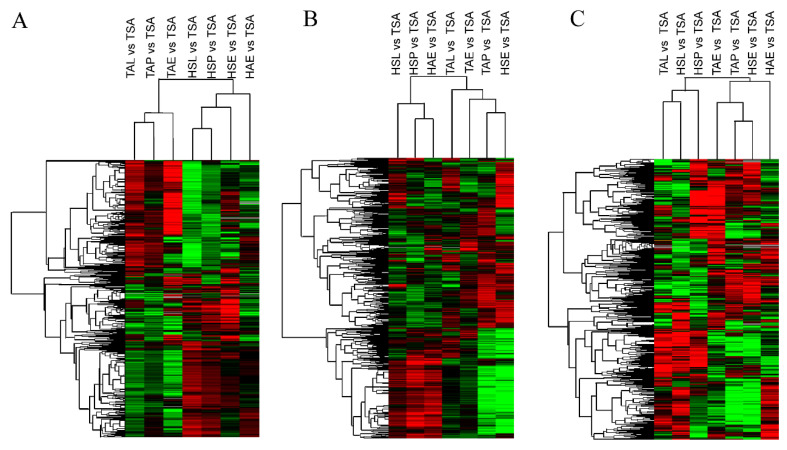
Hierarchical Clustering of DE mRNAs among seven group comparisons. (**A**) The hierarchical clustering result of DE mRNAs in Hypothalamus. (**B**) The hierarchical clustering result of DE mRNAs in pituitary. (**C**) The hierarchical clustering result of DE mRNAs in ovary.

The hierarchical clustering analysis was used to compare the expression patterns of DE mRNAs (Figure 3) for identifying key genes. The results showed that sheep of the same breed showed similar expression patterns in the same tissue. In the hypothalamus and pituitary, the expression level of small tail Han sheep during spring estrus was slightly different from that of other periods (Figure 3A,B). However, there was no significant difference in the clustering of ovarian expression levels (Figure 3C).

### 3.3. Enrichment Analysis of DE mRNAs

#### 3.3.1. Enrichment Analysis of DE mRNAs of Three Tissues at Different Period

**Figure 4:**
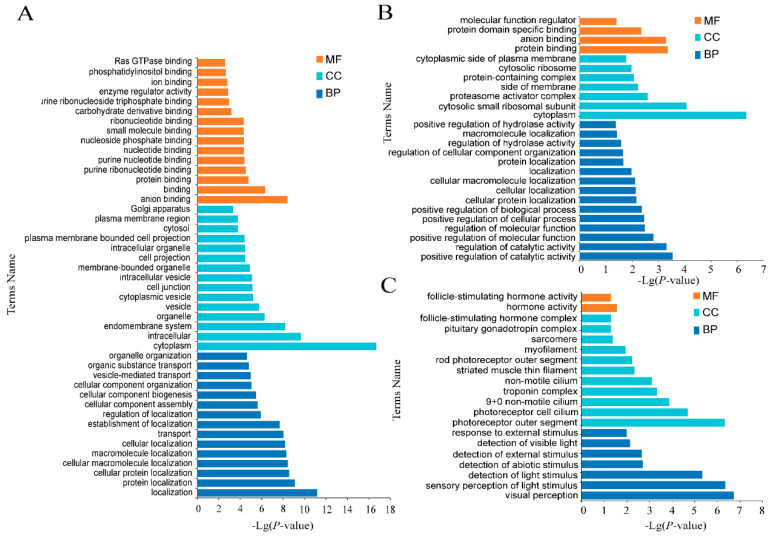
Enrichment analysis of hypothalamus mRNAs. (**A**) Enriched GO terms of luteal stage, (**B**) Enriched GO terms at proestrus stage, (**C**) Enriched GO terms at estrus stage. Different bar chart colors represent different items. Dark blue represents BP, light blue represents CC, and orange indicated MF.

**Figure 5:**
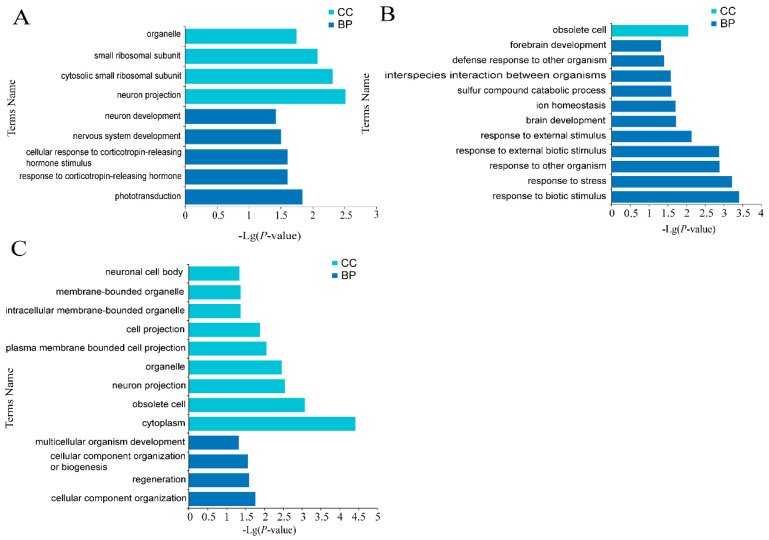
Enrichment analysis of pituitary mRNAs. (**A**) Enriched GO terms of luteal phase, (**B**) Enriched GO terms of proestrus, (**C**) Enriched GO terms of estrus. Dark blue represents BP, light blue represents CC, and orange indicated MF.

**Figure 6:**
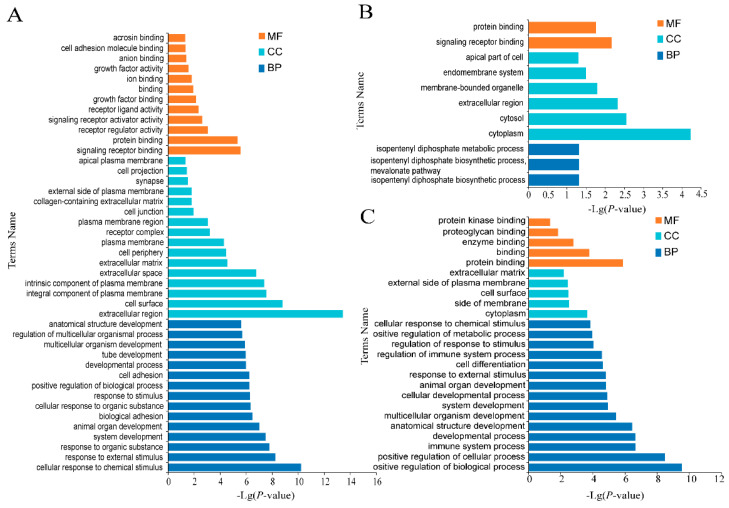
Enrichment analyses of ovary mRNAs. (**A**) Enriched GO terms of luteal phase, (**B**) Enriched GO terms of proestrus, (**C**) Enriched GO terms of estrus. Different bar chart colors represent different items. Dark blue represents BP, light blue represents CC, and orange indicated MF.

The roles of these DE mRNAs in the regulation of estrus and anestrus in sheep were unclear. To better illustrate the functions of the DE mRNAs in estrus and anestrus stages at the global level, Gene Ontology (GO) and the Kyoto Encyclopedia of Genes and Genomes (KEGG) enrichment was used to annotate the DE mRNAs. The GO term and KEGG term of significant enrichment are shown respectively (Figure 4, Figure 5 and Figure 6, Table 2 and Table S3). GO was used to analyze enrichment terms of DE mRNA in hypothalamus (Figure 4), pituitary (Figure 5) and ovary (Figure 6) during luteal phase, proestrus and estrus, respectively. In the functional enrichment GO analysis of hypothalamus at luteal stage (Figure 4A), biological process (BP) terms mainly focus on cellular protein localization, substance transport, activity regulation and other processes were enriched, while cellular component (CC) entries including vesicle formation, including cytoplasmic vesicle and intracellular vesicle were enriched. Molecular function (MF) terms mainly enriched in nucleic acid formation process, including purine ribonucleotide binding and purine nucleotide binding. Among 27 GO terms significantly enriched in hypothalamic proestrus (Figure 4B), biological process regulation and molecular function regulation were significantly enriched. Similarly, KEGG analysis showed that Phototransduction pathway was also enriched in hypothalamus during proestrus. During the hypothalamic estrus period (Figure 4C), 23 GO entries were significantly enriched which mainly includes the perception of light stimulation and follicle stimulating hormone activity, including sensory perception of light stimulation (BP), visual perception (BP) and follicle-stimulating hormone activity (MF).

Phototransduction and Axon guidance were mainly enriched by KEGG analysis during pituitary luteal phase. The GO terms enriched in the pituitary were mainly classified into BP and CC. The GO terms enriched in the luteal phase (Figure 5A) were mainly categorized to the response process of corticotropin-releasing hormone and the development process of nervous system. The GO items enriched in the pituitary were mainly categorized to the response process of adrenocorticotropin-releasing hormone and the development process of nervous system. The GO items significantly enriched in proestrus (Figure 5B) are mainly categorized into the biological stimulation response process. The significantly enriched GO items during estrus (Figure 5C) are mainly categorized into cell membrane and organelles.

The pathways of BP, CC and MF were significantly enriched in all three stages of ovary. In the luteal phase of ovary (Figure 6A), BP entries were mainly classified into steroid biosynthesis, metabolism, cell proliferation and apoptosis. CC and MF entries mainly focus on the composition of plasma membrane and the binding process of signal receptors. Thirty-three GO entries were significantly enriched in the ovarian proestrus (Figure 6B), mainly in isoprene diphosphate synthesis [16], metabolic process, cytoplasm, signal receptor binding and protein binding. Most of the GO items in ovarian estrus (Figure 6C) are in the BP process, including cell proliferation and apoptosis, immunoregulation, and hematopoietic regulation. CC and MF entries mainly includes plasma membrane and protein binding. Biosynthesis, regulation of cytoskeleton and focal adhesion were significantly enriched in ovary.

#### 3.3.2. GO Enrichment Analysis of Specific mRNAs in HSE in Small Tail Han Sheep

**Figure 7:**
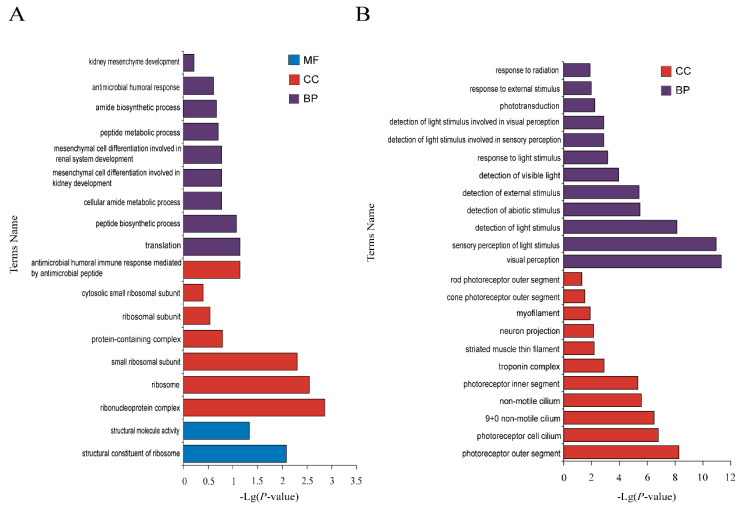
The functional enrichment analysis of specific genes expressed in the hypothalamus of Small Tail Han Sheep. (**A**) Enriched GO terms of luteal phase, (**B**) Enriched GO terms of estrus. Different bar chart colors represent different items. Purple represents BP, red represents CC, and blue indicated MF.

**Figure 8:**
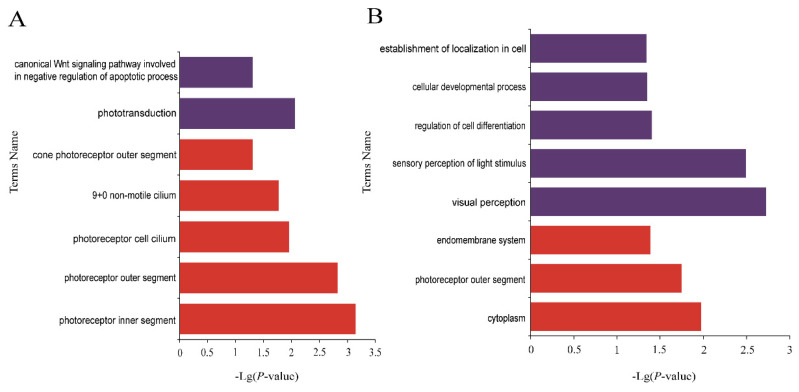
The functional enrichment analysis of specific genes expressed in the pituitary of Small Tail Han Sheep. (**A**) Enriched GO terms of luteal phase, (**B**) Enriched GO terms of estrus. Different bar chart colors represent different items. Purple represents BP, red represents CC, and blue indicated MF.

**Figure 9:**
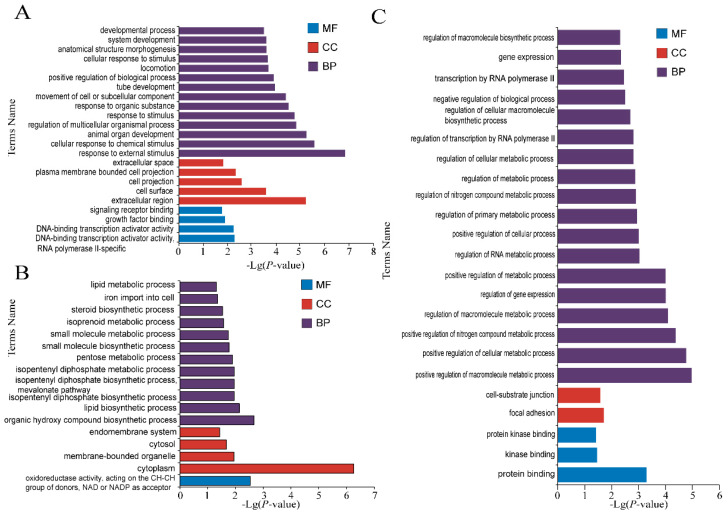
The functional enrichment analysis of specific genes expressed in the ovary of Small Tail Han Sheep. (**A**) Enriched GO terms of luteal phase, (**B**) Enriched GO terms of proestrus, (**C**) Enriched GO terms of estrus. Different bar chart colors represent different items. Purple represents BP, red represents CC, and blue indicated MF.

In order to sift through the important genes regulating year-round estrus, a comparison was made between Tan sheep (seasonal estrus) and STH (year-round estrus), and the specific expressed genes in small tail Han sheep were retained for functional enrichment analysis. That is, the DE mRNAs only different expressed in STH not different expressed in Tan sheep. A total of 1292 DE genes from the three tissues were annotated by thousands of GO terms (Figure 7, Figure 8 and Figure 9, Tables S3 and S4). In the luteal phase, there were 63 GO pathways that were significantly enriched by means of GO enrichment analysis (*p*-value < 0.05), including 44 biological processes (BP), 13 cellular components (CC), and six molecular functions (MF). In the proestrus, only 17 GO pathways were significantly enriched in ovarian DE genes (*p*-value < 0.05). There were 81 GO pathways with remarkably enriched genes in estrus (*p*-value < 0.05). The items obvious enriched by different genes in hypothalamus (Figure 7B) and pituitary (Figure 8B) during estrus are mainly related to visual perception, photoreceptors, photostimulation response, visible light detection, etc. The items enriched in ovaries in connection with metabolism, regulation, mitosis and signal transduction. The pathway of significant enrichment from the luteal stage to estrus stage, interestingly enough, shows the transition from the preparation of material synthesis to the photosensitive pathway. The pathways of significantly enriched in luteal phase of ovary were focused on cell communication, small molecule synthesis and cell development, while the biosynthesis of steroids, the synthesis of sex hormones and other related pathways appeared at the proestrus stage. During estrus stage, cell metabolism and regulation of macromolecular biosynthesis gene expression were significantly enriched.

### 3.4. Protein-Protein Interaction (PPI) Network Construction and Analysis

**Figure 10:**
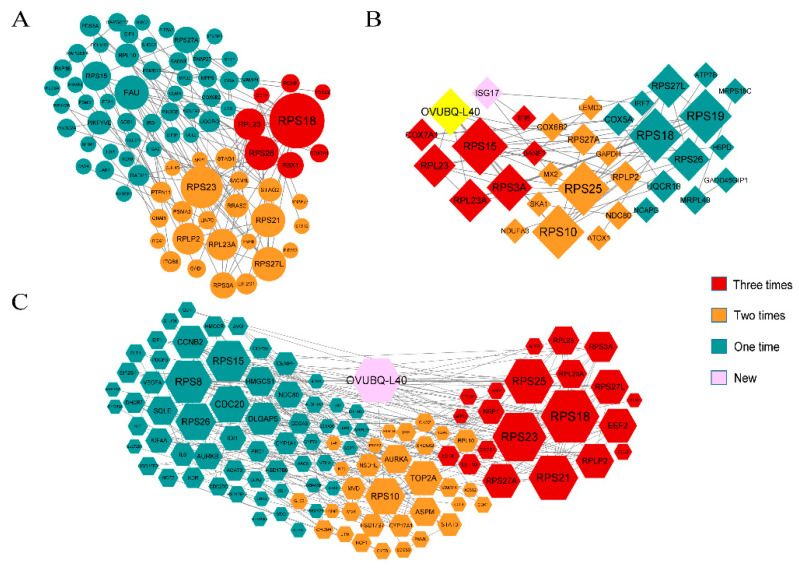
Overview of the protein-protein network related with three tissues (**A**) The interaction network of hypothalamus differential protein with circulars (**B**) The interaction network of pituitary differential protein with diamonds (**C**) The interaction network of ovary differential protein with hexagons. The size of shape indicates Degree. Different colors correspond to the number of participating stages.

**Figure 11:**
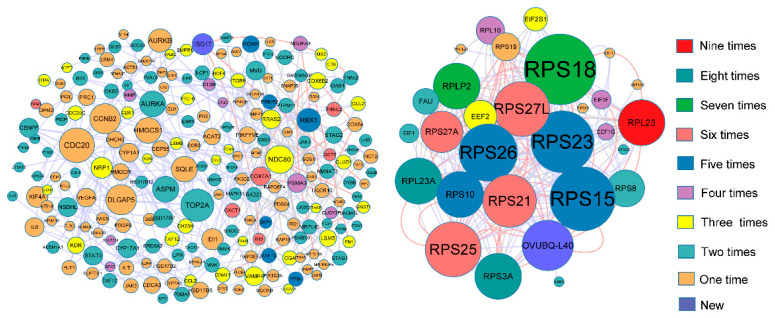
The interaction network of differential protein with three tissues. The size of shape indicates Degree. Different colors correspond to the number of participating stages.

The DE proteins of the three tissues at different stages were uploaded to the String database (https://string-db.org/ accessed on 30 June 2021). Based on the protein information contained in the database, protein interaction networks were constructed respectively (Figure 10, Table S5). RPS and RPL protein families are at the core of the interaction network in the hypothalamus, pituitary and ovary, among which RPS18 has the highest degree of connection with other proteins. In addition, after summarizing total DE proteins in three tissues, it was founded that RPS and RPL families were closely connected to each other, and it was independent (Figure 11).

**Figure 12:**
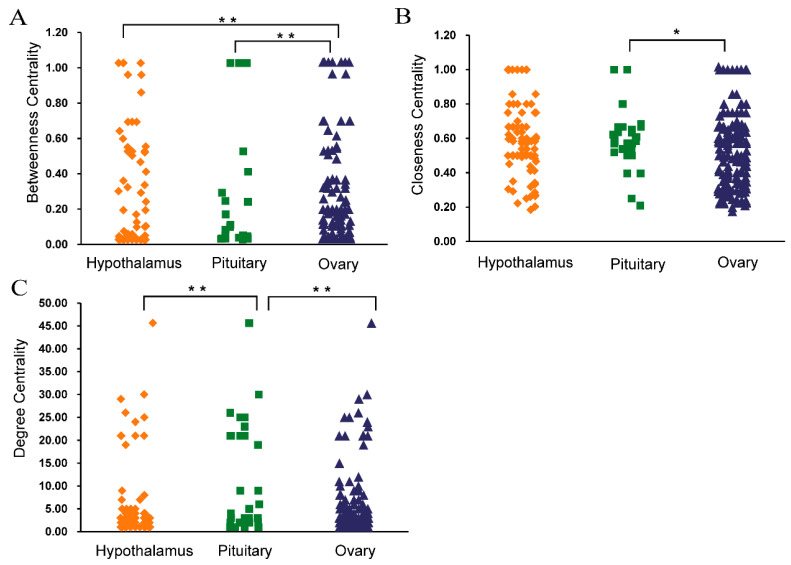
The differences in the betweenness, closeness, and degree centrality among hypothalamus, pituitary and ovary (**A**) The scatterplot of betweenness centrality. (**B**) The scatterplot of closeness centrality (**C**) The scatterplot of degree centrality. The significant results with a *p*-value lower than 0.05 are given one asterisk (*). The extremely significant results with a *p*-value lower than 0.01 are given two asterisks (**).

The betweenness, closeness centrality, and network degree of the top 20 proteins are shown in Table 3. In addition to *RPS* and *RPL* family. There are genes (*CDC20*, *TOP2A*, *EEF2*, *DLGAP5*, *CCNB2*) in the top 20 besides *RPS* and *RPL* protein families. As DE protein, RPL23 was identified at the three stages of hypothalamus, pituitary and ovary. The topological features of this total network were assessed by a built-in Network Analyzer tool in Cytoscape software, including betweenness, network degree, and closeness centrality (Table S6). Afterwards, One-way analysis of variance (ANOVA) was used for the difference analysis of three organizational Betweenness, network degree and closeness centrality separately. Hypothalamus and pituitary had significantly higher contents of betweenness centrality than ovary. (*p* < 0.01) (Figure 12A). The pituitary of closeness centrality was obviously higher than ovary (*p* < 0.05) (Figure 12B). The network degree of pituitary was significantly different between hypothalamus and ovary (*p* < 0.01) (Figure 12C).

## 4. Discussion

Recently, RNA-seq has been widely used as a common method in biology and medicine to analyze gene expression and discover novel RNA [18]. Additionally, RNA-seq were also used for studying single-cell gene expression, translation and RNA structure. RNA plays an important role in the regulation of biomolecules and biological processes, such as splicing and translation, which involve the interaction of RNA with various proteins or other RNA molecules [19]. In this study, RNA-seq technology was used to analyze the differences of gene expression in three tissues of STH and Tan sheep at different stages. However, STH is a year-round breed of estrus, while tan sheep is seasonal estrus. The result of differential gene expression is more likely to reflect the difference between STH and tan sheep, which is valuable for future research to determine the main genes or new genes affecting sheep reproduction.

It has long been known that there were seasonal variations in the reproductive capacity in sheep. They usually start their estrous cycle when there is less sunlight, and end it when there is more sunlight. The optical signal is a significant factor in controlling the seasonal reproduction of animals [20]. These seasonal changes were overcome by regularly exposing ewes to short periods of light during normal dark hours [21]. The hypothalamus [22], pituitary [23], and ovary [24,25] have been thought to form the axis of the regulatory ring controls ovulation, and almost all reproductive hormones are secreted by the hypothalamus-pituitary-ovarian (HPO)axis [26,27]. Most of these hormones are proteins, and they are regulated in synthesis and secretion mainly at the transcriptional and post-transcriptional levels [23]. The HPO axis communicates with each other through hormonal signals among these three key organs [28]. The hypothalamic secreted gonadotropin-releasing hormone (GnRH) stimulates pituitary gonadotropin synthesis and releases gonadotropin, follicle-stimulating hormone (FSH), and LH. Then, these hormones stimulate the growth and maturation of follicles and the expulsion of oocytes [29,30].

### 4.1. Pathway Enrichment Analysis

In this study, cluster analysis showed that the activation of differentially expressed genes was distinct in different tissues of the two breeds sheep at different time points. It was particularly important to find out the causal genes or significant enrichment pathways of year-round estrus in sheep during anestrus season. Based on the RNA-seq data, the differentially expressed genes in three tissues of STH were analyzed. A large number of light-sensing pathways are enriched during the estrus stage in pituitary. During proestrus in the ovary, pathways were enriched on Valine, leucine and iso-leucine biosynthesis, lipid biosynthetic process, isopentenyl diphosphate biosynthetic process, and mevalonate pathways. In 2020, Bai et al. found that valine and leucine were consumed in the follicular fluid of inactive cows to supplement energy [31]. Many metabolites are found in non-estrous follicular fluid. Therefore, we suspected that similar changes in follicular fluid molecules may affect ovarian activity in sheep. In addition, upstream regulatory organs of ovary, hypothalamus and pituitary, affected by the light cycle and synchronize with rhythm, can stimulate downstream ovary to start estrus and enter into breeding cycles. As for the seasonal difference between the Small Tailed Han sheep and the Tan sheep, the results of GO and KEGG analysis showed that photoreceptor pathway was enriched at the luteal phase in hypothalamus of STH, while it was not enriched at the same stage of Tan sheep (Figure 4A and Figure 7A). Researchers have illustrated a conserved neuroendocrine pathway which govern seasonal breeding. The retina receive light stimulation to produce optic nerve impulse which was transmitted to the suprachiasmatic nuclei (SCN) of the hypothalamus. SCN being main circadian clock in animals can transmit signals to the pineal gland causing pulsatile release of melatonin (MEL) at night. Thus, photoperiod through melatonin is the main driver to initiate the signal transmission, and MEL provided an internal endocrine indicator for external photoperiod [32,33,34]. It can be speculated that the genes categorized into photoperiod transmission or annual rhythm were very likely selected under domestication and artificial selection. From our gene expression results, pathways related to photoreceptor and phototranduction were highly activated in non-seasonal breeding breed like STH, thus the neuroendocrine transmission between photoperiod and downstream reproductive activity may be mobilized more frequently than it was in Tan sheep.

### 4.2. Candidate Genes for Seasonality in Sheep

As mentioned above, the photoperiod can initiate signal transmission through melatonin, while MEL provides an internal endocrine index of photoperiod. Downstream melatonin receptor can receive melatonin rhythmic secretion signal, which causes a series of biological reactions, and then causes pulsatile release of gonadotropin-releasing hormone (GnRH). In hypothalamus, GnRH can regulate seasonal reproductive cycle through a series of reproductive hormones in hypothalamic-pituitary-gonadal axis (HPGA). Therefore, its members need to cooperate closely on the Hypothalamus-pituitary-ovarian (HPO) axis and mobilize several related genes and proteins joint photoperiod to make it consistent with endogenous reproductive activities.

For this study, a total of 2569, 2704 and 4156 genes were significantly differentially expressed in Hypo-thalamus, pituitary and ovary, respectively. By means of GO, KEGG and PPI analysis, several key genes identified by expression profiling were enriched in prolificacy processes, such as ornithine decarboxylase 1 (*ODC1*) [35], prolactin releasing hormone (*PRLH*) [36], crystallin β B2 (*CRYBB2*) [37], ribosomal protein S6 (RPS6) [38], SMAD family member 5 (*SMAD5*) [39], and associated with Biological rhythms such as opsin 1, short wave sensitive (*OPN1SW*) [40], F-box and leucine rich repeat protein 7 (FBXL7) [41], tryptophan hydroxylase 1 (*TPH1*) [42], while other genes may be involved in the follicular development or control of ovulation. For example, genes enriched in protein binding pathways include melanocyte inducing transcription factor (*MITF*) [43] in ovary at estrus stage, and 24-dehydrocholesterol reductase (*DHCR24*) [44] in pituitary. Presence of the various polyamines and other related factors (ODC1, etc.) were demonstrated by immunohistochemistry in the hypothalamus of rats during the estrous cycle. ODC1 proteins were mainly cytoplasmic and localized in the medial preoptic area (MPA) of the hypothalamus particularly during the proestrus, estrus and diestrus phases [35]. ODC1 is expressed in ovarian granules and follicular membrane cells and may be involved in the regulation of ovarian follicle formation and luteinization [45,46]. Follicular formation is involved in cell proliferation, therefore ODC1 may play a key role in follicular formation and luteinization by promoting cell proliferation and differentiation. Both estrogen and progesterone play key roles in follicular development [47,48]. *CRYBB2* is mainly expressed in ovarian granulosa cells, which may affect the proliferation function of ovarian granulosa cells. Moreover, follicular development is a complex process requiring vigorous proliferation of granulosa cells [49]. Loss of *CRYBB2* results in inhibition of cell cycle progression in granular cells [37]. Notably, the mutation of *CRYBB2* may cause congenital nuclear cataracts in humans [50]. Whether there are similar mutations in sheep that affect its photosensitive function is not known. ODC1 and *CRYBB2* were identified in hypothalamus and pituitary at estrus stage, respectively, and we hypothesized that they may play the same role in sheep. SMAD protein is the main intracellular signal transductor of bone Morphogenetic protein (BMP) family receptors [51], and *SMAD5* mediates the intracellular signal transduction of BMP [52,53,54,55]. *SMAD5* was differentially expressed in the uterus of mice, indicating that the importance for estrous cycle. SMAD5 is a major protein involved in regulating uterine function during the estrous cycle [39]. Therefore, we hypothesized that *SMAD5* in sheep may be associated with signal transduction between hypothalamus and ovary. *TPH1* is one of the major genes involved in melatonin (MLT) synthesis and metabolism. The TPH1 protein catalyzes the production of tryptophan into serotonin, providing a substrate for the synthesis of melatonin *TPH1*. The expression of *TPH1* gene was consistent with melatonin synthesis [55,56].Thus, reducing the activity of *TPH1* can be achieved by changing the synthesis efficiency of 5-hydroxytryptamine, and it may cause melatonin synthesis to become less efficient. All those may make animals less sensitive to light signals and eventually lead to the decrease or disappearance of seasonal breeding in mammals [42]. In addition, Liu et al. found that there has a missense mutation, T865G in *TPH1*. It affects the function of TPH protein, which may contribute to the genetic potentiality of Mongolian sheep to year-round estrus [57]. Similarly, in addition to *TPH1*, there are also a large number of genes related to light signal perception in our study. We speculate that these genes may regulate downstream genes derived by annual rhythm, and finally exhibit reproductive cycles.

In summary, it is believed that the key DE mRNAs in the hypothalamus, pituitary and ovary are directly or indirectly involved in hormone-related activities related to reproduction. However, this study is limited to bioinformatics analysis, and the specific functional mechanism during estrous cycles in sheep breeds needs further experimental verification.

## 5. Conclusions

During the estrus period of STH, biosynthesis of macromolecules, synthesis of steroid hormones, follicle, cell development and other related genes remained active. The hypothalamus and pituitary are particularly sensitive to light stimulation during estrus. Light is thought to be a main driver in the initiation of estrous cycles in sheep which was consistent with previous research. The transcriptomic profiling of STH and Tan sheep were presented which can provide a new perspective and reliable data for further study on seasonal estrus trait in sheep.

## Figures and Tables

**Table 1:** The number of DE mRNAs of two breed sheep in hypothalamus, pituitary, ovary in different period.

		Luteal Phase		Proestrus		Estrus		
		TAL	HSL	TAP	HSP	TAE	HAE	HSE
Hypothalamus	Anestrus (TSA)	1068	181	607	87	331	102	193
	Co-DE mRNAs	21		15		0		
Pituitary	Anestrus (TSA)	374	205	640	100	556	170	285
	Co-DE mRNAs	51		28		10		
Ovary	Anestrus (TSA)	676	884	385	606	526	772	307
	Co-DE mRNAs	315		93		52		

**Table 2:** The significantly enriched pathways of KEGG analyzes.

Tissue	Source	Term Name	*p*-Value
Hypothalamus estrus	KEGG	Cardiac muscle contraction	0.0003
		Ribosome	0.0094
		cAMP signaling pathway	0.0144
		Phototransduction	0.0266
Pituitary luteal phase		Axon guidance	0.0063
		Ribosome	0.0279
Ovary Proestrus		Valine, leucine and isoleucine biosynthesis	0.0043
Ovary estrus		Regulation of actin cytoskeleton	0.0070
		Focal adhesion	0.0469
Ovary Luteal phase		Biosynthesis of unsaturated fatty acids	0.0455

**Table 3:** Degree of node from protein-protein interaction network.

Proteins	Degree	Closeness	Betweenness	Class	Proteins	Degree	Closeness	Betweenness	Class
RPS18	45	0.80000000	0.26577395	Hypothalamus Pituitary Ovary	RPLP2	21	0.60869565	0.00530952	Hypothalamus Pituitary Ovary
RPS15	30	0.63636364	0.01884148	Hypothalamus Pituitary Ovary	RPL23A	21	0.59574468	0.00551146	Hypothalamus Pituitary Ovary
RPS23	29	0.70000000	0.07679841	Hypothalamus Ovary	RPL23	21	0.53846154	0.00659906	Hypothalamus Pituitary Ovary
RPS26	26	0.62222222	0.01139418	Hypothalamus Pituitary Ovary	RPS27A	19	0.53846154	0.00659906	Hypothalamus Pituitary Ovary
RPS27L	25	0.60869565	0.00802717	Hypothalamus Pituitary Ovary	RPS8	15	0.58333333	0.00052910	Hypothalamus
RPS25	25	0.68292683	0.05605774	Pituitary Ovary	CDC20	12	0.66666667	0.13099227	Hypothalamus
RPS21	24	0.66666667	0.03142804	Hypothalamus Ovary	TOP2A	11	0.73076923	0.20673907	Hypothalamus
OVUBQ-L40	23	0.65116279	0.08473492	Pituitary Ovary	EEF2	11	0.67857143	0.09162490	Hypothalamus
RPS3A	21	0.58333333	0.00893936	Hypothalamus Pituitary Ovary	DLGAP5	10	0.60869565	0.07270322	Hypothalamus
RPS10	21	0.80000000	0.26577395	Pituitary Ovary	CCNB2	10	0.65517241	0.14796018	Hypothalamus

## Data Availability

Not applicable.

## Supplementary Materials

The following are available online at www.mdpi.com/xxx/s1/, Table S1: Summary of raw reads. Table S2: List and statistics of differential genes. Table S3: Enriched GO and KEGG terms of three tissues. Table S4: Enriched GO terms of specific genes expressed in Small Tail Han Sheep. Table S5: The data of protein-protein interaction. Table S6: The data of betweenness, network degree, and closeness centrality.

## Abbreviations

RNA-seq	RNA sequencing
SD	short-day
LD	long-day
HPG	hypothalamus-pituitary-gonad
GnRH	gonadotropin releasing hormone
LH	luteinizing hormone
FSH	follicle stimulating hormone
STH	Small Tail Han sheep
TSA	Tan ewes in spring at anestrous stages
TAL	Tan ewes in autumn at luteal phase
TAP	Tan ewes in autumn at proestrus stage
TAE	Tan ewes in autumn at estrus stage
HSL	STH ewes in spring at luteal phase
HSP	STH ewes in spring at proestrus
HSE	STH ewes in spring at estrus stage
HAE	STH ewes in autumn at estrus stage
GO	Gene Ontology
DE mRNAs	Differentially Expressed mRNAs
Co-DE mRNAs	Co-Differentially Expressed mRNAs
PPI	protein-protein interaction
BP	biological process
CC	Cellular component
MF	Molecular function
CDC20	cell division cycle 20
TOP2A	DNA topoisomerase II α
EEF2	eukaryotic translation elongation factor 2
DLGAP5	DLG associated protein 5
CCNB2	cyclin B2
RPL23	ribosomal protein L23
HPO	hypothalamus-pituitary-ovarian
HPGA	hypothalamic-pituitary-gonadal axis
ODC1	ornithine decarboxylase 1
PRLH	prolactin releasing hormone
CRYBB2	crystallin β B2
RPS6	ribosomal protein S6
SMAD5	SMAD family member 5
FBXL7	F-box and leucine rich repeat protein 7
TPH1	tryptophan hydroxylase 1
MITF	melanocyte inducing transcription factor
DHCR24	24-dehydrocholesterol reductase
MPA	medial preoptic area
BMP	bone Morphogenetic protein
MLT	melatonin
